# Hand Injuries in the Oil Fields of Brunei Darussalam

**DOI:** 10.5704/MOJ.1303.016

**Published:** 2013-03

**Authors:** Pramod Devkota, Shiraz Ahmad

**Affiliations:** Department of Orthopaedics and Trauma Surgery, Suri Seri Begawan Hospital, Kuala Belait, Brunei Darussalam; Department of Orthopaedics and Trauma Surgery, Suri Seri Begawan Hospital, Kuala Belait, Brunei Darussalam

## Abstract

Hands are essential organs and their agility and dexterity are vital to our daily lives. In the present study, we analysed 107 patients who presented at the local hospital with hand injuries sustained in the oil fields, oil industries and related employment sectors from the surrounding regions. All the patients were male and the mean age was 37.89 years (range, 21-61y). Forty-seven (43.93%) patients had simple cut injuries, 14 patients (13.08%) had tendon injuries, 13 patients (12.14%) had amputation of the digit (30.84%) had bone fractures (including 20 (66.66%) open fractures). Only 19 (17.75%) patients were admitted in hospital for further treatment. Ninety-one (85.04%) patients injured within one year of employment and 57(53.27%) patients were not satisfied with instructions and orientation before starting their job. Hand injury is one of the most common injuries in the oil industry and overtime work further increases incidence of this injury.

**Key Words:**

Hand injury, trauma, oil field, digital fractures

## Introduction

Injuries are the fifth common cause of death among men and the sixth most common among women^1^. Injuries to the hand are among the most frequent injuries, constituting between 6.6% and 28.6% of all injuries and 28% of injuries to the musculoskeletal system^2^. Such injuries occur mainly during industrial activities and about one-third of all work-related wounds are hand injuries, with consequences ranging from deformity to mortality^3^.

Manual skilled, semiskilled and unskilled workers typically sustain more hand injuries that can result in high treatment costs, a lengthy treatment period and often permanent disability^4^. The economic, social, and physical impact of permanent or temporary disability on the loss of productive working hours is a heavy burden on the local community^5^.

Here we report our experience of the hand injuries that occurred in the oil fields of Brunei Darussalam.

## Materials and Methods

This is a descriptive observational retrospective study conducted at the Department of Orthopaedics and Trauma Surgery of Suri Seri Begawan Hospital, Kuala Belait, Brunei Darussalam. All oil field injuries are brought to our hospital for the treatment, as it is the largest healthcare institution in the vicinity of the oil fields and second largest general hospital in the country. From January 2010 to April 2012, we reviewed medical records of 107 patients who were treated for hand injuries sustained in the oil fields. All patients were referred to an orthopaedic surgeon through the Accident and Emergency Department or directly referred by oil field medics. We obtained all necessary approvals from the institutional ethics committee for this study.

Only patients with hand injuries suffered in the oil fields were included in this study. Hand injuries from other causes were excluded. Patients were interviewed during the follow up period or via telephone about workload, and pre-employment orientation and training. Demographic characteristics, length of employment, type of injuries and treatment given were analysed. We used the student t-test for statistical analysis.

## Results

All patients were male and the mean age was 37.89 years (range, 21- 61 years). All patients said they were injured while wearing protective gloves provided by the company. Forty-seven patients (43.93%) had simple cut injuries of the hand, 14 patients (13.08%) had tendon injuries and 13 patients (12.14%) had amputation of digits. Thirty-three (30.84%) patients had bone fractures, of which 20 (66.66%) were open fractures. Only 19 patients (17.75%) were admitted to the hospital for inpatient treatment. Ninety-one (85.04%) patients were injured within one year of employment. Of note, 57 patients (53.27%) were not satisfied with their pre-employment instructions and orientation before starting work, while 52(48.6%) believed that this was a truly accidental injury. A majority of patients (51.4%) were injured while working overtime.

**Fig. 1:**
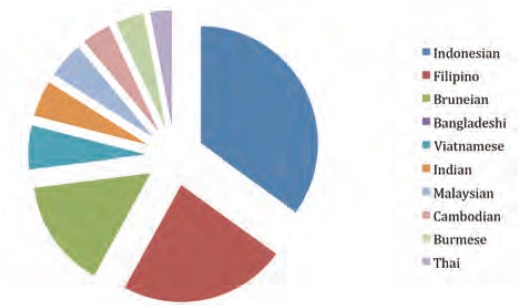
: Incidence of hand Injuries among the nationalities. Of 107 patients presenting with hand injury the following is the breakdown by nationality: Indonesian, 35 patients (32.72%); Filipino, 23 patients (21.40%); Bruneian, 15 patients (14.01%); Vietnamese, 6 patients (5.61%); Indian, 5 patients (4.67%); Malaysian, 5 patients (4.67%); Cambodian, 4 patients (3.74%), Burmese patients (3.74%); Thai, 3 patients (2.81%).

Student test (T-test) was done to analyze between the true accident and less training incidence and found not statistical significance (P>0.05). Statistical significant (P<0.05) was found while analyzing the incidence of injury within one year of job time and after one year of job time. Among the nationals, Indonesians were the first followed by Filipino and Bruneian to get the injury (Figure 1).

Eighty-seven (81.31%) patients injured their dominant hand; most of the injured individuals were between the age of 31 and 40 years (Table I); most (68.22%) patients were unmarried and a majority reported completion of primary education (Table II). Phalangeal fractures were the most common fractures among the bony injuries. Extensor tendons injuries were more common than flexor tendon injuries and the little finger distal end amputation was the most common amputation.

## Discussion

Industrial hand injuries are a common occurrence. Although mortality from hand injury is rare, it is among the biggest contributors to morbidity in oil field workers. Understanding the predisposing factors for hand injury is important but it is extremely difficult to prevent. In the present study, all presenting hand injury patients from the oil fields and oil related industries were men similar to other reports of high incidence of hand injuries in men. This is not surprising as men comprise the majority of the labour force in the industrial market^5^,^6^,^7^,^8^.

Simple cut injury comprised a majority of the hand injuries in this study, and most of the injured patients were 20 – 40 years with only a primary education. This is consistent with the other reports from around the world^2^,^9^,^10^,^11^.

Many hand injury patients were foreign expatriates had who come to work in the oil fields of Brunei Darussalam. Indonesian and Filipino workers sustained the most hand injuries followed by Bruneian workers. Those with less than one year of oil field experience were injured more, but not to a statistically significant degree. Other studies also showed similar rates of hand injuries among expatriates and immigrants^5^,^12^,^13^,^14^.

A majority of patients were injured while working overtime period were not satisfied with their pre-employment orientation and instructions; this finding was also consistent with results from other studies^5^,^7^,^15^,^16^,^17^. In the study, a majority of patients (51.4%) laid blame for the injury on inadequate training and slightly less than half of the patients said it was truly an accident, though there was not a significant difference in this regard. Proper instructions and orientations may reduce the incidence of hand injuries. These results also point out the need for proper rest periods as fatigue led to some of the injuries.

A limitation of this study is its retrospective nature and short time period studies. A prospective study with a longer study period is necessary to gain deeper knowledge about the working conditions and appropriate measures to prevent hand injuries.

**Table I:**
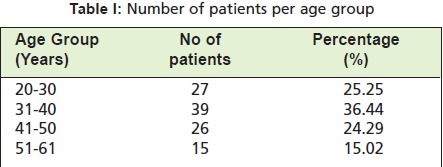
: Number of patients per age group

**Table II:**
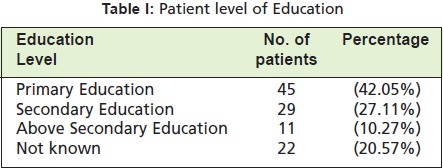
: Patient level of Education

**Fig. 2:**
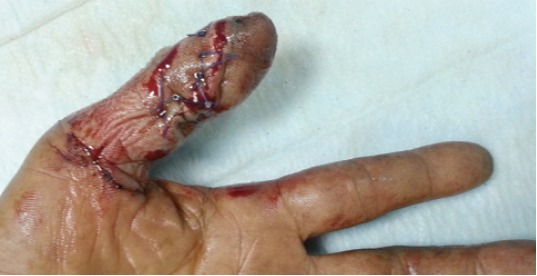
: Photograph showing crush injury of a left thumb in a 40- year old man.

## Conclusion

Hand injuries are one of the common injuries in the oil fields; such injuries may be reduced by appropriate preemployment orientations and instructions. A lack of experience and overtime work render oil field workers susceptible to hand injury.
